# Infant difficult behaviors in the context of perinatal biomedical conditions and early child environment

**DOI:** 10.1186/1471-2431-12-44

**Published:** 2012-04-11

**Authors:** Giedre Sirvinskiene, Nida Zemaitiene, Apolinaras Zaborskis, Egle Markuniene, Roma Jusiene

**Affiliations:** 1 Institute for Health Research, Lithuanian University of Health Sciences, Academy of Medicine, Eiveniu str. 4, LT-50009 Kaunas, Lithuania; 2 Department of Health Psychology, Lithuanian University of Health Sciences, Academy of Medicine, Kaunas, Lithuania; 3 Department of Neonatal Diseases, Lithuanian University of Health Sciences, Academy of Medicine, Kaunas, Lithuania; 4 Department of General Psychology, Vilnius University, Vilnius, Lithuania

## Abstract

**Background:**

Problems experienced within the first year of an infant's life can be precursors of later mental health conditions. The purpose of this study was to examine the frequency and continuity of difficult behaviors in infants at 3 and 6 months of age and the associations of these difficulties with biomedical and psychosocial factors.

**Methods:**

This study was a part of an ongoing prospective birth-cohort study. Study participants were 189 uniparous mothers and their full-term newborns. The index of infant difficult behavior was constructed. This index was then associated with the following factors: delivery mode, newborn function after birth, maternal emotional well-being, risk behavior, subjective evaluation of the quality of the relationship of the couple, and attitudes toward infant-rearing.

**Results:**

Common difficult behaviors, including crying, sleeping and eating problems, were characteristic for 30.2% of 3 month old and for 22.2% of 6 month old full-term infants. The expression of infant difficult behaviors at the age of 3 months increased the likelihood of the expression of these difficulties at 6 months by more than 5 times. Factors including younger maternal age, poor prenatal and postnatal emotional well-being, prenatal alcohol consumption, low satisfaction with the couple's relationship before pregnancy, and deficiency of infant-centered maternal attitudes towards infant-rearing increased the likelihood of difficult behaviors in infants at the age of 3 months. Low maternal satisfaction with the relationship of the couple before pregnancy, negative emotional reactions of both parents toward pregnancy (as reported by the mother) and the deficiency of an infant-centered maternal attitude towards infant-rearing increased the likelihood of infant difficult behaviors continuing between the ages of 3 to 6 months. Perinatal biomedical conditions were not related to the difficult behaviors in infants.

**Conclusions:**

Our study suggests that early onset of difficult behavior highly increases the risk for the continuation of difficult behavior during infancy. In general, the impact of prenatal psychosocial environment on infant behavior decreases from the ages of 3 to 6 months; however, some prenatal and preconceptional psychosocial factors have direct associations with the continuity of difficult behaviors through the first half-year of an infant's life.

## Background

Studies of mental health condition precursors underscore the importance of the infancy period for a child's long-term adjustment [1,2]. Infant problems experienced during the first year of life can contribute to emotional and behavioral problems later in childhood. Infant problems can also have a negative impact on maternal well-being. Excessive crying, sleeping, or feeding problems during infancy, often referred to as infant regulatory problems, are found in approximately 20% of infants. Infant regulatory problems increase the likelihood of deficits in preschool adaptive behavior and social skills [3], as well as increase the risk of childhood behavioral problems [4]. Studies of mental health in children point out the importance of prenatal events and the postnatal environment of the child [5]. Prematurity, serious medical illness, infant temperament, parental psychopathology, infant-caregiver attachment, marital quality and interactions, poverty, social class, and family violence are among the major risk conditions to harmonious child development [6]. Numerous studies indicate that the disturbances in the infant-caregiver relationship are the key risk mechanisms in early child psychopathology [7]. However, the interaction of these risk factors is complex and multidirectional. Persistence of infant behavioral problems contributes to maternal depression, parental stress, and subsequent child behavioral issues [8]. Maternal depression is associated with a significant reduction in secure attachment of the infant, raising the likelihood of avoidant behavior and disorganized attachment of the infant [9]. Furthermore, maternal depression increases vulnerability during later development of a child's psychopathology and affects emotional, behavioral, psycho-physiological, and cognitive functioning [10]. Some evidence suggests that developmental problems in infants can be influenced by prenatal maternal mental health, as well as by medical conditions (e.g. birth complications) alone [11].

Some studies indicate that early biomedical circumstances surrounding the birth of a child affect parental behavior, infant behavior, and later postnatal development. Indicators of a more stressful delivery are linked to more frequent crying and fussing in the infant, and to more difficulty regulating the infant's behavior [12]. The mode of delivery and analgesia used during birth is associated with maternal and fetal endocrine stress responses [13]. Several types of analgesia given to the mother during labor may interfere with the newborn's spontaneous breast seeking and breast-feeding behaviors, and may increase the newborn's crying [14]. However, the possible impact of medical conditions surrounding birth on later child mental health and behavior is not clear and research integrating psychosocial and biomedical risk factors of infant and child problem behavior requires further investigation.

Unlike subjects in other research that focuses on infants of various risk groups, the subjects of this study were full-term newborns. To carry out an in-depth investigation of the roots of difficult behavior it is also important to clarify the mechanisms of such behavior, as well as the reasons behind it within normal development and conditions. The aims of our study are to examine the frequency and continuity of infant difficult behaviors at 3 and 6 months of age, and to determine the biomedical and psychosocial factors linked with early infant difficulties. In this paper, we focus on the common difficult behaviors manifested by infants, which include crying, sleeping, and eating problems.

## Methods

### Study design and sample

This study is the initial part of an ongoing prospective birth-cohort study started in 2009. The research was performed with the approval of the Kaunas Regional Biomedical Research Ethics Committee (No. P1-143/2007). The study participants were uniparous mothers who gave birth to full term newborns (≥ 37 weeks of gestation) in the Hospital of the Lithuanian University of Health Sciences, Kaunas Clinics. This is a Baby Friendly Hospital, where the medical staff strictly follows the principles of early breastfeeding initiation and rooming-in. After vaginal delivery, newborns stay on close skin-to-skin contact with their mothers. After caesarean section delivery, newborns stay in the nursery room until their mothers are transported to the intensive care ward, and within two hours, the newborn is transported to the mother for breastfeeding.

This study consisted of three stages. The first stage was performed in the clinics, and included the collection of medical data about delivery and newborn function, as well as the collection of data from questionnaire surveys for mothers during their stay. Written informed consent was obtained from women who participated in the study together with their infants. The questionnaires were given to mothers during the second or third day after childbirth; the new mothers were asked to answer them at their own convenience. The first stage involved data received from 546 mothers. The second stage was performed three months postpartum, during which women were asked to complete mail-in questionnaires. A total of 242 participants responded to the request and completed the questionnaires. The third stage of research was carried out six months postpartum, during which women were once again asked to complete the mail-in questionnaires. Completed questionnaires were received from a total of 261 respondents. We selected the data of those women who participated in all three stages of the study, a total 189 participants, for the analysis. Their mean age was 28.38 (SD = ± 5.70).

We also checked whether the demographic data differed between women who participated in all three study stages, and were included in analysis (participants), and those who were excluded from analysis (non-participants, did not participate in all three surveys). The analysis indicated no differences according to mean age, place of residence, or marital status. However, a marked difference was discovered with regards to higher education (college or university degree) among participants: 138 participants (70.4%) had received higher education, and only 199 of the non-participants (57.8%) had received higher education (*χ*^2 ^= 8.95, p = 0.011).

## Measures

### Perinatal biomedical variables

The hospital medical staff collected biomedical information about newborns and their mothers. *Delivery mode and medication administration *used during childbirth were included in the data analysis. Newborns born via cesarean section were compared with vaginally-born newborns. The vaginal deliveries were then grouped into oxytocin administration and epidural anesthesia deliveries, and interventions and deliveries with no medication administration.

#### Birth weight

According to birth weight and gestational age, newborns were divided into three groups: normal birth weight (≥ 10 and ≤ 90 percentiles), small for gestational age (SGA) neonates (birth weight < 10 percentile for their gestational age) and large for gestational age (LGA) neonates (birth weight > 90 percentile for their gestational age) according to Lithuanian national birth weight standards [15].

*Apgar scores 1 and 5 minutes after birth *were used to evaluate newborn functioning.

*The neonatal neurological and adaptive capacity *was assessed at 2 hours, and at 24 hours, after birth by a neonatologist using the Neurological and Adaptive Capacity Scale (NACS) [16]. The NACS is based on twenty criteria, each of which is given a score of 0, 1, or 2 based on whether the response to testing that criterion is absent or grossly abnormal (0), mediocre or slightly abnormal (1), or normal (2). The maximum possible score is 40. These criteria assess five general areas: 1) adaptive capacity; 2) passive tone; 3) active tone; 4) primary reflexes; and 5) alertness, crying, and motor activity. According to the authors of the NACS scale, scores of 35 and above indicate good newborn neurological and adaptive capacity.

### Perinatal psychosocial variables

During the first study stage, on the 2^nd^-3^rd ^day postpartum, psychosocial data about prenatal and short postpartum period were collected. The participants completed the *Prenatal environment questionnaire*, whose questions covered demographics, pregnancy planning, emotional reactions toward the conception, the relationship with the husband or partner, tobacco use and alcohol consumption, and emotional experiences during pregnancy and postpartum. All prenatal variables were scored retrospectively during this stage.

The *demographic characteristics *of the participants were highlighted using questions about maternal age, education, and family structure.

The participants were asked whether the pregnancy was planned or not-planned in order to evaluate *pregnancy planning*. Next, *the emotional reactions toward pregnancy *were evaluated by the questions 'Which statement best reflects your reaction toward pregnancy?' and 'Which statement best reflects your husband or partner's reaction toward your pregnancy?' with possible answers 'happy', 'conflicting feelings', 'upset', or 'other'. In the analysis, the answers were divided into two groups: the first group as the positive reaction, and the second group as the ambivalent or negative reactions, including conflicting feelings, upset feelings, or other negative emotional reactions.

*The quality of the couple's relationship *was subjectively evaluated by mothers using the Likert type scale, rating from 'very bad relationship' (1 point) to 'very good relationship' (5 points). Mothers were asked to evaluate the relationship with husband or partner before and during pregnancy.

*Tobacco and alcohol use during pregnancy *was evaluated by the questions 'Did you smoke cigarettes during pregnancy?' and 'Did you consume alcohol during pregnancy?' The possible answers were as follows: 'not at all,' 'several times during the whole pregnancy,' 'once or several times a month,' 'once or several times a week,' and 'everyday.' The women who reported not smoking during pregnancy were categorized as 'nonsmokers', those who reported smoking several times during whole pregnancy, or several times a week or a month were defined as 'non-regular smokers'; those who had smoked everyday were categorized as 'regular smokers'. According to alcohol consumption, women were divided into two groups: those who reported prenatal alcohol consumption and those who did not report alcohol consumption during pregnancy.

To evaluate *stressful and traumatic experience during pregnancy*, women were asked whether or not they had experienced any stressful and traumatic events during pregnancy.

*Negative emotional experiences during pregnancy *were evaluated by questions on how often during pregnancy they were experienced emotions such as irritability/bad temper, feeling low and feeling nervous; mothers were given the possibility to choose one of five statements for each emotion: 'almost daily,' 'more often than once a week,' 'almost every week,' 'almost every month' and 'rare or never,' evaluated from 1 to 5 points. The total sum score was used to evaluate negative emotional experience, where lower sum score indicates more often experienced negative emotional state.

*Depressiveness 2 to 3 days postpartum *was measured by the Edinburgh postnatal depression scale [17]. The Lithuanian version of Edinburgh postnatal depression scale was used in this study [18]. Cronbach's alpha for this measure was 0.81.

### Psychosocial variables measured 3 months postpartum

Three months postpartum, during the 2^nd ^stage of the study, the participants completed the *Infant development and social environment questionnaire*. This survey asked questions regarding parental social support, the relationship of the couple, maternal emotional state, breastfeeding, postpartum maternal attachment, and maternal attitudes toward child rearing. Women were also asked if the father of the infant is living with her and the child, and if he helps with childcare.

*The quality of the couple's relationship, stressful and traumatic experiences*, as well as *negative emotional experience during three months postpartum*, and *depressiveness 3 months postpartum *were measured using the same questions as during the 1^st ^stage.

*Breastfeeding the baby *was also included in the analysis. Women were asked if they are breastfeeding their infants or not.

*Maternal attitudes toward caring for an infant *were assessed using the Infant-rearing attitudes and beliefs scale [19]. The measure attempted to gauge the extent to which individuals hold "infant-centered" versus "parent-centered" views regarding infant care. The opposite ends of the spectrum might also be conceived of as "rigid" versus "flexible" infant-rearing practices. For example, endorsing the statement "Babies should be fed on a fixed time schedule" is considered parent-centered and rigid, whereas endorsing the statement "Babies should be fed whenever they want" is considered infant-centered and flexible. A high score on this measure indicates an infant-centered, flexible approach to parenting. Respondents were asked to indicate on an 8 point scale, ranging from *very strong agreement *to *very strong disagreement*, how much they endorse the viewpoint expressed in each of the eight statements. The statistical analysis showed that Cronbach's alpha for this measure was 0.74.

### Dependent variable

Infant difficult behaviors were selected as a dependent psychosocial variable in this study. It was assessed at the ages of 3 and 6 months using *Women's perception of infant's difficult behaviors *scale [20]. The original scale consisted of 10 infant difficulties. However, because of low component score values for some items, we excluded three from the scale and used results delivered from answers to the remaining 7 items. Mothers checked whether (1) or not (0) the baby had experienced each of the following behaviors in the last month: (1) prolonged or frequent diarrhea or constipation, (2)pronounced lack of interest in being fed or active refusal to eat, (3) excessive demand to be fed, (4) frequent waking and crying at night, (5) frequent and intense crying generally, (6) noticeable stiffening, turning away, or crying when picked up or handled, and (7) pronounced clinging when picked up or intense crying when put down. The internal consistency reliability (Cronbach α) coefficients of the used scale were 0.64 and 0.60 correspondingly at the ages of 3 and 6 months.

### Statistical analysis

Basic and advanced statistics procedures of the Statistical Package for Social Sciences (SPSS) for Windows version 15.0 software package were used to conduct data analysis. The first analyses included descriptive statistics and primarily frequencies; this provided an understanding of the distributions of the respondents' demographic, social, psychological and medical characteristics during the pregnancy and childbirth period, as well as infant's health, mental development, and behavioral problems. We applied the *χ*^2 ^test and Z test, where appropriate, to assess the differences in the prevalence of characteristics between different groups of respondents. A measure of agreement (*kappa*) was used to assess the consistency of responses on an infant's problem behavior between the ages of 3 and 6 months. In testing statistical hypotheses, a *p*-value of less than 0.05 was considered significant.

In order to assess infant difficult behavior in general, 7 items of the *Women's perception of infant's difficult behaviors *scale were combined into one derivative variable, labeled "Index of Infant's Difficult Behaviors" (IIDB). It was not the simple sum of scores in responses to each of 7 questions of the scale, but rather a linear combination of them with different weights. The values of weights in this linear combination were estimated from the total data set by the SPSS Factor Analysis procedure requesting a single factor, and then that single factor (IIDB) for each record (factor score value) was calculated and saved. The method of factors extraction was based on *principal component *analysis [21]. The weights of the variables in their linear combination, as indicators of a variable's involvement in a factor, reflect the partial variable variances in the factor variance. The percentage of the total variance of all 7 items of the scale explained by the IIDB was 34.9 and 33.26 at the infant's ages of 3 and 6 months correspondingly (Table 1). The values for IIDB appeared to be distributed within the range of -0.50 and 6.48; its mean and standard deviation estimates, as follows by definition, were respectively 0 and 1. Quantitative values of the IIDB were re-coded into two categories. The first category included the negative and zero values of the index that were typical of infants whose mothers reported a few difficult behaviors; the second category included positive values of the index that were common for infants with a higher rate of difficult behaviors. These categories were labeled as "low rate of difficult behaviors" and "high rate of difficult behaviors" correspondingly.

**Table 1:** Component Score Values of the *Women's perception of the infant's difficult behaviors *Scale estimated at the infant's age of 3 and 6 months

Items of the Scale	At the age of 3 months	At the age of 6 months
Prolonged or frequent diarrhea or constipation	0.15	0.08

Pronounced lack of interest in being fed or active refusal to eat	0.18	0.25

Excessive demand to be fed	0.21	0.22

Frequent waking and crying at night	0.27	0.20

General frequent and intense crying	0.31	0.29

Noticeable stiffening, turning away, or crying when picked up or handled	0.31	0.28

Pronounced clinging when picked up or intense crying when put down	0.20	0.33

Eigen value	2.44	2.33

Percent variance	34.9	33.26

Extraction Method: Principal Component Analysis.

The last analyses included binary univariate logistic regression analysis procedures. Odds ratios (OR) and 95% confidence intervals (CI) by adjusting data for infant's gender were calculated in order to analyze the associations between different demographic, social, psychological and medical variables and IIDB. For this purpose, the sample was split into two groups according to low or high rates of infant's difficult behaviors (negative or positive IIDB) at the infant's ages of 3 months, 6 months and at both 3 and 6 months. Calculations were performed separately for each of the three sample groupings.

## Results

### Infant difficult behaviors prevalence and continuity

The statistical analysis showed that the different items of Index of Infant Difficult Behaviors at 3 and 6 months varied according their weights. The strongest weights at 3 and 6 months included items such as infant crying (Table 1). We thus concluded that infant crying is among the items that explain the greatest amount of variance, explained by the factor of infant behavioral difficulties.

The Index of Infant Difficult Behaviors illustrated that a high rate of difficult behaviors was characteristic for 30.2% of 3 month old and for 22.2% of 6 month old full-term infants. 13.2% of infants had a high rate of difficult behaviors at ages of 3 and 6 months. According to the data presented in Table 2, the onset and stability of difficulties differ according to the infant's age. Some of the difficult behaviors, such as excessive demand to be fed, decrease from the age of 3 to 6 months, while others, for example waking and crying at night, become more prevalent in infants over this span. Pronounced clinging when picked up or intense crying when put down could be described as the most persistent difficulties from 3 to 6 months.

**Table 2:** Changes in infant difficult behaviors among the ages of 3 and 6 months

Infant's difficult behaviors	Proportion of infants (%)			Measure of agreement *kappa*
		
	High rate of difficult behaviors at the age of 3 months	High rate of difficult behaviors at the age of 6 months	High rate of difficult behaviors at both 3 and 6 months	
Prolonged or frequent diarrhea or constipation	11.0	13.9	2.3	0.072

Pronounced lack of interest in being fed or active refusal to eat	6.4	7.6	2.3	0.284***

Excessive demand to be fed	11.1	5.3	1.8	0.154*

Frequent waking and crying at night	4.7	18.1	3.5	0.252***

General frequent and intense crying	2.9	3.5	0.6	0.155*

Noticeable stiffening, turning away, or crying when picked up or handled	6.5	6.5	1.8	0.222**

Pronounced clinging when picked up or intense crying when put down	13.4	9.9	4.7	0 0.323***

Index of infant difficult behaviors^†^	30.2	22.2	11,1	0.266***

* p < 0.05; ** p < 0.01; *** p < 0.001.

^† ^dichotomized into negative and positive values, which correspond to low and high rates of difficult behaviors.

Logistic regression analysis proved that a strong association exists between difficult behaviors at 3 and 6 months: the likelihood of difficult behaviors at the age of 6 months increased more than five times (OR = 5.29; 95% CI 2.53-11.00; p < 0.001) if such problems were reported at the age of 3 months. The prevalence of a high problem rate among 6-month-old infants was greater if these infants were characterized as having a greater amount of problems at the age of 3 months, compared with 3-month-old infants with a low problem rate (43.9% and 12.9% respectively). However, a remarkable decrease of problem behavior after 3 months was also observed; greater than half (56.1%) of the infants with problem behavior at 3 months stopped by 6 months of age (Table 3).

**Table 3:** Continuity of infant difficult behaviors from 3 to 6 months of age

Infant difficult behaviors	Proportion of infants (%)		Total
		
	Low rate of difficult behaviors at the age of 6 months	High rate of difficult behaviors at the age of 6 months	
Low rate of difficult behaviors at the age of 3 months	115 (87.1)	17 (12.9)	137(100,0)

High rate of difficult behaviors at the age of 3 months	32 (56.1)	25 (43.9)	52(100,0)

Total	147 (77,8)	42 (22,2)	189(100,0)

### Associations of infant difficult behavior with biomedical and psychosocial factors

The associations between infant behavioral difficulties at different ages and demographic, biomedical and psychosocial factors are presented in Table 4.

**Table 4:** Univariate association among different prenatal and postnatal variables and infant's difficult behaviors at the age of 3 months, 6 months and at both 3 and 6 months: odds ratio (OR) with 95% confidence interval (CI)^†^

		At the age of 3 months	At the age of 6 months	Both at 3 and 6 months
		**OR (95% CI)**	**OR (95% CI)**	**OR (95% CI)**

***Demographic characteristics***				

*Maternal age*: ≤ 25	64 (34.0)	2.58 (1.15-5.77)*	2.25 (0.95-5.36)	2.64 (0.91-7.59)
25-30 years	62 (33.0)	1	1	1
≥ 31	62 (33.0)	1.72 (0.76-3.91)	1.19 (0.48-2.96)	1.05 (0.32-3.47)

*Family structure*: Marriage	156 (83.0)	1	1	1
Cohabitation	25 (13.3)	1.71 (0.71-4.10)	1.20 (0.44-3.27)	1.95 (0.65-5.84)
Single	7 (3.7	1.81 (0.39-8.52)	2.43 (0.51-11.59)	2.80 (0.50-17.77)

*Education*: Secondary or lower	55 (27.3)	1.05 (0.53-2.07)	1.30 (0.62-2.74)	1.41 (0.59-3.37)
Higher (college or university)	133 (70.7)	1	1	1

*Infant's gender*: Boy	107 (56.6)	1.20 (0.64-2.25)	1.72 (0.84-3.53)	1.43 (0.60-3.41)
Girl	82 (43.4)	1	1	1

First child	103 (54.8)	1	1	1
Have older children	85 (45.2)	0.90 (0.86-1.70)	1.22 (0.60-2.47)	0.92 (0.39-2.15)

***Perinatal biomedical variables***				

*Delivery mode:*				
Vaginal delivery	131 (69.7)	1	1	1
Cesarean section	57 (30.3)	1.72 (0.89-3.33)	1.03 (0.49-2.18)	1.09 (0.44-2.70)

*Medications used during vaginal delivery*:				
No medications	67(54.9)	1	1	1
Epidural anesthesia	19 (15.6)	2.13 (0.73-6.19)	1.12 (0.34-3.66)	1.71(0.46-6.33)
Oxytocin	22 (18.0)	0.48 (0.19-2.18)	0.90(0.28-2.89)	0.64(0.13-3.20)
Epidural and oxytocin	14 (11.5)	0.81 (0.20-3.27)	0.91 (0.22-3.76)	1.10(0.21-5.75)

*Birth weight: *Normal	154 (81.5)	1	1	1
SGA	9 (4.8)	0.71 (0.14-3.55)	1.04(0.21-5.32)	0.88(0.10-7.44)
LGA	26 (13.8)	1.53 (0.64-3.62)	0.83(0.29-2.36)	1.24(0.39-3.97)

*Apgar scores 1 minute after birth: *9-10	43 (75.7)	1	1	1
8 or lower	46 (24.3)	0.56 (0.26-1.24)	1.78 (0.84-3.80)	1.24(0.48-3.18)

*Apgar scores 5 minutes after birth*: 9-10	174 (92.1)	1	1	1
8 or lower	15 (7.9)	0.15 (0.02-1.18)	0.52(0.11-2.43)	0.00(0.00-0.00)

*The NASC 2 hours after birth: *Good	73 (41.5)	1	1	1
Bad/not good	103 (58.5)	1.08 (0.56-2.07)	0.74(0.36-1.50)	0.87(0.37-2.04)

*The NASC 24 hours after birth: *Good	122 (71.3)	1	1	1
Bad/not good	49 (28.7)	1.26 (0.62-2.56)	1.69 (0.79-3.59)	1.09(0.422.85)

***Perinatal psychosocial variables***				

Pregnancy planed	134 (74.0)	1	1	1
Not planed	47 (26.0)	1.33 (0.70-2.92)	1.51 (0.68-3.33)	2.00 (0.77-5.21)

*Maternal reactions toward conception: *Positive	149 (78.8)	1	1	1
Negative/ambivalent	40 (21.2)	1.66 (0.77-3.55)	1.70 (0.77-3.75)	3.66(1.47-9.17)**

*Paternal reactions towards conception:*				
Positive	159 (84.6)	1	1	1
Negative/ambivalent	29 (15.4)	2.15 (0.95-4.85)	1.79(0.74-4.33)	4.16(1.61-10.76)**

*Couple's relationship before pregnancy: *Good	112 (60.2)	1	1	1
Average or bad	74 (39.8)	2.59(1.35-4.94)**	1.50 (0.74-3.06)	3.06(1.25-7.48)*

*Couple's relationship during pregnancy: *Good	114 (61.3)	1	1	1
Average or bad	72 (38.7)	1.87 (0.99-3.53)	1.11(0.55-2.25)	1.89(0.81-4.42)

*Prenatal smoking:*				
Non-smoking	158 (87.8)	1	1	1
Non-regular smoking	14 (7.8)	0.99 (0.30-3.34)	0.63 (0.13-2.96)	0.53 (0.07-4.25)
Regular smoking	8 (4.4)	2.46 (0.59-10.28)	2.20 (0.50-9.81)	2.23 (0.42-11.86)

*Prenatal alcohol us*e: No	105 (58.0)	1	1	1
Yes	76 (42.0)	1.94 (1.01-3.72)*	1.70 (0.82-3.51)	2.30 (0.95-5.58)

*Stressful and traumatic experience during pregnancy*:				
No	150 (80.6)	1	1	1
Yes	36 (19.4)	2.63 (1.23-5.59)*	1.89(0.83-4.32)	1.52(0.55-4.19)

*Negative emotional experience during pregnancy*: Rare	92 (51.1)	1	1	1
Often	88 (48.9)	2.41 (1.24-4.67)*	1.46 (0.71-2.99)	1.30(0.55-3.06)

*Depressiveness 2-3 days postpartum*: Low	162 (85.7)	1	1	1
High	27 (14.3)	0.95 (0.38-2.33)	0.72 (0.25-2.05)	0.46(0.10-2.09)

***Psychosocial variables measured 3 months postpartum***				

*Father of the infant living together: *Yes	148 (90.8)	1	1	1
No	15 (9.2)	2.83 (0.96-8.33)	2.03(0.63-6.49)	2.52(0.72-8.77)

*Father helps in childcare:*				
Yes	138 (86.8)	1	1	1
No	21 (13.2)	1.73 (0.68-4.43)	1.57(0.28-8.73)	1.41(0.43-4.62)

*Couple's relationships during the 3 months postpartum*: Good	101(59.8)	1	1	1
Average or bad	68 (40.2)	1.97 (0.99-3.93)	1.63 (0.74-3.63)	2.74(0.96-7.79)

*Stressful/traumatic experience at 3 month postpartum: *No	143 (82.7)	1	1	1
Yes	30 (17.3)	1.56 (0.69-3.53)	0.52 (0.17-1.62)	0.64(0.18-2.29)

*Negative emotional experience during the 3 months postpartum:*				
Rare	91 (53.8)	1	1	1
Often	78 (46.2)	2.28 (1.18-4.40)*	1.04(0.50-2.15)	1.75(0.73-4.20)

*Depressiveness 3 months postpartum: *Low	164 (86.8)	1	1	1
High	25 (13.2)	1.98 (0.84-4.70)	1.70(0.67-4.30)	2.33(0.82-6.59)

*Breastfeeding: *Yes	134 (75.3)	1	1	1
No	44 (24.7)	1.14 (0.54-2.39)	1.33(0.61-2.95)	2.00(0.80-4.97)

*Maternal attitudes towards infant-rearing*:				
Child oriented, flexible	122 (79.7)	1	1	1
Parent oriented, rigid	31 (20.3)	2.38 (1.06-5.36)*	1.84 (0.76-4.48)	3.31 (1.25-8.74)*

* p < 0.05; ** p < 0.01; *** p < 0.001.

^†^adjusted by infant's gender

#### Demographic variables and perinatal biomedical factors

Younger maternal age was associated with increased likelihood of difficult behavior at the age of 3 months. However, characteristics such as marital status, maternal and parental education, infant's gender, and birth order were not related with infant difficulties. We observed no associations between infant difficulties with delivery mode, medication administered during labor and newborn functioning.

#### Psychosocial factors

As follows from Table 4, factors reflecting the emotional state of the mother during prenatal and postnatal periods are of great importance. The presence of infant difficulties at 3 months of age was related to poor evaluation of relationships with the husband or partner before pregnancy, prenatal alcohol use, stressful or traumatic events, and frequent negative emotional experience both during pregnancy and the first months after childbirth, as well as maternal attitudes toward infant-rearing, consisting of more rigid and parent-centered views. Not one of these variables was related to infant difficult behaviors at the age of 6 months. The obtained data show that the persistence of difficult behaviors between the ages of 3 to 6 months increased significantly if the mother reported negative emotional reactions toward pregnancy, if she stated that the father expressed negative emotional reactions towards conception, if she evaluated the her relationship before pregnancy poorly, and if she lacked a infant-centered maternal attitude toward infant rearing.

## Discussion

Our research revealed that the early manifestation of infant difficult behaviors increases the risk for continuation of problems during the first half-year of infancy. If a child demonstrates difficult behaviors at the age of 3 months the likelihood that these difficulties will persist through the age of 6 months increases by more than 5 times. However, the study demonstrated that the various forms of difficult behaviour vary with infant age. There is growing evidence of the persistence of problems experienced by very young children [22-24]. At the same time, it should be noted that infants during their first half-year still have the possibility to recover from such difficulties, as more than half of the infants characterized as having problems at age of 3 months were no longer characterized by their mothers as problematic at 6-months-of-age.

The present study showed that during the first half year of infancy, various psychosocial factors differ significantly with regard to their risk for development and persistence of infant difficult behaviors. According to the data, demographic and prenatal psychosocial factors including maternal age, emotional well-being and risk behaviors highly increased the risk for difficult infant behavior, but only at the age of 3 months. By the age of 6 months, the aforementioned factors became less important.

In this study, we had the opportunity to evaluate what factors are related to the persistence of infant behavioral difficulties between 3 and 6 months of age. The stability of infant difficulties was related to negative maternal and paternal emotional reactions (as reported by the mother) toward conception, low satisfaction with the couple's relationship before pregnancy, and rigid parent-centered maternal attitudes towards infant rearing. This suggests a strong significance of parent-related and family-related characteristics for an infant's development. Young families, especially those with poor relationships, low readiness for parenthood, and deficient child-oriented attitudes should therefore receive early prevention programs, which focus on teaching infant-rearing skills.

In general, it can be stated that the direct significance of some aspects of prenatal history (such as maternal emotional well-being) decrease during the first half year of infancy, while aspects of the postnatal child environment (such as maternal attitudes toward infant-rearing) become more significant. This could be explained by the complex interplay between a child and his or her environment over time, and the confounding effect of risk factors. Bidirectional effects of the child and of the environment are highlighted in transactional models [25] and are well-documented in various representative studies of experimental, quasi-experimental, and naturalistic design [26]. However, our study also suggests that some negative aspects of prenatal history (such as poor quality of a couple's relationship before pregnancy and negative or ambivalent reactions toward conception) could potentially have long-lasting effects, increasing the risk of continuity of infant difficult behaviors. It can be assumed that these outward factors could also be interrelated with maternal emotional well-being and produce a combined effect along with the mother's long-lasting negative emotional state.

The results of this study also document the importance of maternal prenatal stress and emotional well-being in the emergence of infant difficult behaviors at 3 months of age, although it was not related with later infant problems. The effects of prenatal anxiety/stress on a child's difficulties developing in cognitive, behavioral, and emotional ways are documented in numerous studies [27]. The prenatal stress associations with ADHD symptoms, externalizing problems, anxiety [28], and sleep disturbances [29] have already been highlighted by other researchers. Symptoms of depression, pregnancy-related anxiety, parenting stress, and job strain during pregnancy were also found to be associated with excessive infant crying [30]. We did not find associations of the maternal depression and infant behavior, suggesting that maternal depression may have not direct associations with infant behavior. Some authors have found that maternal depression only explained infant problem behavior in high-risk samples; neither maternal depression nor medical complications in pregnancy predicted problem behaviors within low risk group categories [11].

Our study endorsed the importance of the paternal role in a child's development from the very beginning of pregnancy. Based on the study data, the quality of a couple's relationship before pregnancy and paternal emotional reactions toward conception could have long-lasting effects on infant behaviors and may be of greater significance than the quality of the couple's relationship later in time. Despite growing research interest and recognition of the importance of the father in a child's development, data on the influence of the fathers' role during the early stages of pregnancy and infancy are still lacking. Little attention is given to the relationships of couples before pregnancy; numerous studies exist about the effect of a couple's relationship on offspring development after childbirth or during pregnancy. It is proposed that marital conflict has an impact on the emotional arousal of children and the regulation of emotion and behavior within children [31]. Data has shown, emotional security regarding the marital relationship mediates relations between marital conflict and child adjustment [32]. However, some available data suggest that a poor relationship between the couple during early stages of pregnancy is linked with poor maternal emotional health, health problems, and poor newborn birth weight [33]. A poor relationship between the couple before pregnancy, and the father's negative emotional reactions towards pregnancy, may also be very important factors influencing the emotional well-being of the pregnant mother, later family functioning, and childrearing.

The results revealed the significance of both maternal and paternal emotional response towards conception in respect of infant difficult behaviors. Negative emotional response to the fact that mother became pregnant increases the likelihood of continuation of infant difficult behaviors by about 4 times. Some earlier studies indicate that infants whose conception was unintended by their father are at an elevated risk for adverse health events [34]. Fathers who did not want the pregnancy have been found less likely to exhibit paternal warmth following the birth [35]. Some studies provide evidence that pregnancy unintendedness in women is associated with some disadvantages for prenatal child development, as women whose pregnancies were unintended were found to be more prone to unhealthy behaviors during pregnancy (cigarette smoking and insufficient vitamin intake) [36]. However, a systematic review assessing the effects of unintended pregnancy (mostly studies classifying pregnancies as wanted, mistimed and unwanted) on the health of infants, children, and parents indicates that evidence on impact of unintended pregnancy on child outcomes is mixed and limited. The reviewed studies did not find the effects of pregnancy intention on maternal reports of child health, activity level, and overall development to be evident. Furthermore, if the effects of unwanted and mistimed pregnancies on child development were found, they mostly diminished once family-environment characteristics were included in the model [37]. Thus, other authors underscore the importance of measuring not only how intentional pregnancy was (whether wanted, mistimed, etc.), but also the attitudes and feelings towards pregnancy [38]. In our study, the emotional reactions of the mother and father towards woman conception were significant factors related with infant behavioral difficulties, while the same could not be said about the fact if pregnancy had been planned or not. These results also emphasize the importance to distinguish between pregnancy planning or intendedness, and pregnancy acceptance. The pregnancy intendedness mostly highlights maternal attitudes before conception, while pregnancy acceptance reflects emotional and cognitive response to the pregnancy after conception.

The study indicated that the continuation of infant difficult behaviors between the ages of 3 and 6 months is highly related to postpartum maternal attitudes toward infant rearing. Infants of mothers expressing more parent-centered and rigid attitudes towards infant-rearing had nearly three times of a greater risk for continual behavioural difficulties in comparison with mothers who expressed more flexible and infant-centered attitudes. Other studies provide evidence that mothers' self-reported attitudes correspond with their child rearing behaviors [39]. The parental attitudes toward child rearing are also reported to be related with the parental response to infant crying. Parents characterized to have infant-centered child rearing attitudes had been found to respond to crying at an earlier point, to express greater sympathy, and to perceive crying as urgent [19]. It has been found that infant fussing and crying was related with unresponsive maternal attitudes and behavior [40]. The maternal emotional reactions to crying with anxiety or anger pose risk for subsequent attachment insecurity [41]. The maternal attitudes are changeable factors, and the possibilities to reduce its negative effect on a child's development should be taken into account within various preventive programs.

However, we are considering that factors such as the emotional reactions towards conception and attitudes towards infant rearing are interrelated; perhaps they can be seen as a reflection of the parental acceptance of the child and preparedness to integrate him into the family system. Lack of emotional acceptance and preparedness at this stage might have a later effect on attachment to the child. Studies inspired by attachment theory highlight that the feelings and attitudes individuals have about parent-child relationships, even before they become parents, are predictive of the subsequent infant-mother attachment pattern and the emotional quality of their future parenting behaviors [42]. However, the possible impact of parental emotional reactions toward pregnancy, as well as attitudes toward motherhood or parenthood, is lacking in the literature and requires greater attention.

Several limitations need to be addressed regarding the present study. Our decision to follow the development of full-term newborns (≥ 37 gestation weeks) could be mentioned not only as an advantage, but also a limitations of our study. Since our study was conducted only among low risk infants, the sample size was limited. The second limitation of the study was that all prenatal measures were collected post-natally during the first days after childbirth. The special emotional state of a recently delivered woman could influence responses to the questionnaire. In our study, all information about independent variables (psychosocial factors) and the dependent variable (infant difficult behaviors) were collected from mothers' reports. It was assumed that mothers acted as primary caregivers of infants, predisposing them to greater access to information about infant behavior than other family members. However, the fact that mothers were the only informants in our study may poorly reflect the real circumstances regarding infant behavior and family life; this is another important limitation of this study. Factors such as maternal personality and emotional state after childbirth could significantly influence the reports. This limited the possibility of obtaining more objective data. It is also important to note that information about the fathers' emotional reactions towards pregnancy, as well as the evaluation of the couple's relationship, was also obtained from mothers and could reflect more of the woman's evaluations about her husband/partner than the real feelings and emotions of the fathers. The results of our study, indicating important role of fathers' emotions and the parents' relationship quality on infant difficult behaviors, shows that these issues should be analyzed more comprehensively in further research, with an emphasis on collecting reports from fathers as well. Relatively low reliability scores using the Lithuanian version of the *Women's perception of infant's difficult behaviors *scale should also be noted as another methodological weakness of the present study. Thus, further empirical evaluations are needed.

## Conclusions

Our research revealed that early manifestation of infant difficult behaviors increases the risk for the continuity of these problems during the first half-year of infancy. In general, the impact of prenatal psychosocial environment on infant difficult behaviors decreases between the ages of 3 and 6 months. Although, some prenatal and preconception factors, such as a poor relationship between the couple and negative emotional reactions towards conception, are directly associated with the continuity of infant difficult behaviors throughout the infant's first half year of life. The established importance of attitudes towards child rearing suggests that parental education could be effective in various prevention or intervention programs.

## Competing interests

The authors declare that they have no competing interests.

## Authors' contributions

EM and RJ initiated the study. RJ, EM, NZ, GS participated in the design of the study. GS, NZ and AZ participated in the construction of the current data analyses, data management and interpretations: AZ performed the statistical analysis of the data; GS prepared the draft of the current manuscript, and NZ provided important contributions to the manuscript as well. All of the authors were involved in the revision of the manuscript and contributed to the interpretation of the data. All authors read and approved the final manuscript

## Pre-publication history

The pre-publication history for this paper can be accessed here:

http://www.biomedcentral.com/1471-2431/12/44/prepub
